# The Effects of Ketogenic Diet on Athletic Performance: A Systematic Review

**DOI:** 10.7759/cureus.85566

**Published:** 2025-06-08

**Authors:** Maduka Nnadozie, Chibueze Nnonyelu, Omar Makanay, Daniel Hong, Chinomnso Maduka

**Affiliations:** 1 Family Medicine, AtlantiCare Regional Medical Center, Atlantic City, USA; 2 Internal Medicine, AtlantiCare Regional Medical Center, Atlantic City, USA; 3 Information Technology, Maduka Business Solutions, Washington DC, USA

**Keywords:** endurance exercise, keto diet, low carb diet, performance, weight reducton

## Abstract

This systematic review investigates the impact of a ketogenic diet combined with endurance training on adaptive changes in athletes. The study involved a comprehensive literature search across PubMed, Google Scholar, and Ovid databases, as well as the grey literature to find related studies that have been published since December 2013. The inclusion criteria were as follows: performance data before and after a low-fat high-carb (LFHC) ketogenic diet, a clinical trial, and human subjects. Six relevant studies were included after the selection and exclusion processes. The key fitness parameters analyzed included maximum rate of oxygen consumption (VO2max), Respiratory Exchange Ratio (RER), bench press performance, and body mass changes. The changes in performance parameters and body mass showed some consistency across the different studies, including increased VO2max and reduced body mass. Nevertheless, when compared using the t-test, the resulting data could not be found statistically significant, most likely due to the paucity of data. As a result, we concluded that there are some overall effects of the ketogenic diet on the performance and body composition of athletes. Whether or not these effects are beneficial or whether the diet can be utilized in a manner that confers benefit is yet to be determined. There remains a considerable gap in research on the effects of the ketogenic diet on athletic performance.

## Introduction and background

The ketogenic diet has surged in popularity within the fitness community as a potential method for weight loss and, potentially, athletic performance enhancement. A low-carbohydrate, high-fat (LCHF) diet has been proposed to enhance the fat utilization of muscle and the aerobic capacity of endurance athletes, thereby improving their exercise performance [1]. Central to the ketogenic diet is its significant reduction in carbohydrate intake, often as low as 50 g/day, coupled with a higher proportion of calories derived from fats [2]. This shift in macronutrient composition triggers a notable transition in the body's energy utilization, favoring fat oxidation over carbohydrates [3]. Theoretically, this adaptation enables sustained energy levels due to the abundance of fat stores. Yet, much remains to be elucidated regarding the comprehensive effects of ketogenic diets, particularly how the alteration in macronutrient intake influences endurance exercise training and performance, as indicated by parameters like maximum rate of oxygen consumption (VO2max), respiratory exchange ratio, body composition, and overall strength. This study explores the impact of ketogenic diets specifically on endurance training and the potential adaptive responses in athletes. The expectation was to identify a clear shift in the aforementioned parameters in favor of the ketogenic diet as it relates to performance in endurance exercise.

## Review

Methods

Database Search

Using the Preferred Reporting Items for Systematic Review and Meta-Analyses (PRISMA) guidelines, a robust online database search was conducted, including studies published in the past 10 years up to December 2023. To capture the most updated studies while investigating this topic, the final search was conducted in February 2024. The three databases were PubMed, Google Scholar, and Ovid Web of Science online databases. A grey literature search was also conducted on medrxiv.org. The term "ketogenic diet" was searched and yielded 93 results, only one of which was deemed relevant to our topic; 92 articles were excluded because they did not refer to an investigation of the effects of a ketogenic diet on athletic performance. The search strategy was conducted independently by the first and second authors. When contradicting opinions were raised, the studies were discussed among all the publishing authors until a decision was reached. The key terms used to search databases for articles by topic included: Endurance, athletes, runners, swimmers, cyclists, cross country, ketogenic diet, and performance. The full search strategy for the searches conducted was: ((ketogenic) AND (endurance [Title] OR (athletes [Title] OR (swimmers [Title] OR (cyclists [Title] OR (cross country [Title] OR (performance [Title]))*.*

Inclusion and Exclusion Criteria

The search engine filters for PubMed and Ovid Web of Science were set to include only clinical trials involving human subjects aged 18 years or older. Other inclusion criteria were Free full text, English text, Clinical trial/randomized controlled trial, and quantitative data comparing changes before and after ketogenic diet consumption. Articles were included if the title and abstract demonstrated a focus on the topic of performance changes related to the ketogenic diet. Four hundred and forty-five articles that met the inclusion criteria from each database were compiled using Microsoft Excel software. Duplicate articles obtained from searching multiple databases were removed, leaving 420 articles. The results then underwent a two-step screening process. The first step involved reviewing the publication titles for relevance. This step excluded 299 articles, which left 121 articles for qualitative analysis. The 121 articles were divided among the publishers, and the abstracts, along with the article's content, were screened for relevance. Publishers accepted only articles from clinical trials that quantitatively evaluated secondary outcomes of VO2max, respiratory exchange ratio (RER), and body mass as a measure of exercise/physical activity performance metrics in humans while consuming a ketogenic diet, compared to a standard Western diet. Upon completing these steps, six articles remained for data extraction and quantitative analysis. The summary of the selected articles is displayed in Table 1. The second author assessed the quality of the selected studies and their risk of bias using the Cochrane Collaboration’s Risk of Bias Tool (www.cochrane.org).

Statistical Analysis

The selected articles were reviewed to extract the data needed to compare the results from control groups against the results from the treatment groups via t-test analysis. The outcomes measured and compared were the mean VO2max, the mean change in RER, the mean change in bench press, and the mean change in body mass. Each of the selected studies measured and reported at least one of these outcomes; however, none of the studies included all four of the outcomes of interest.

Results

Synopsis of Included Studies

**Figure 1:**
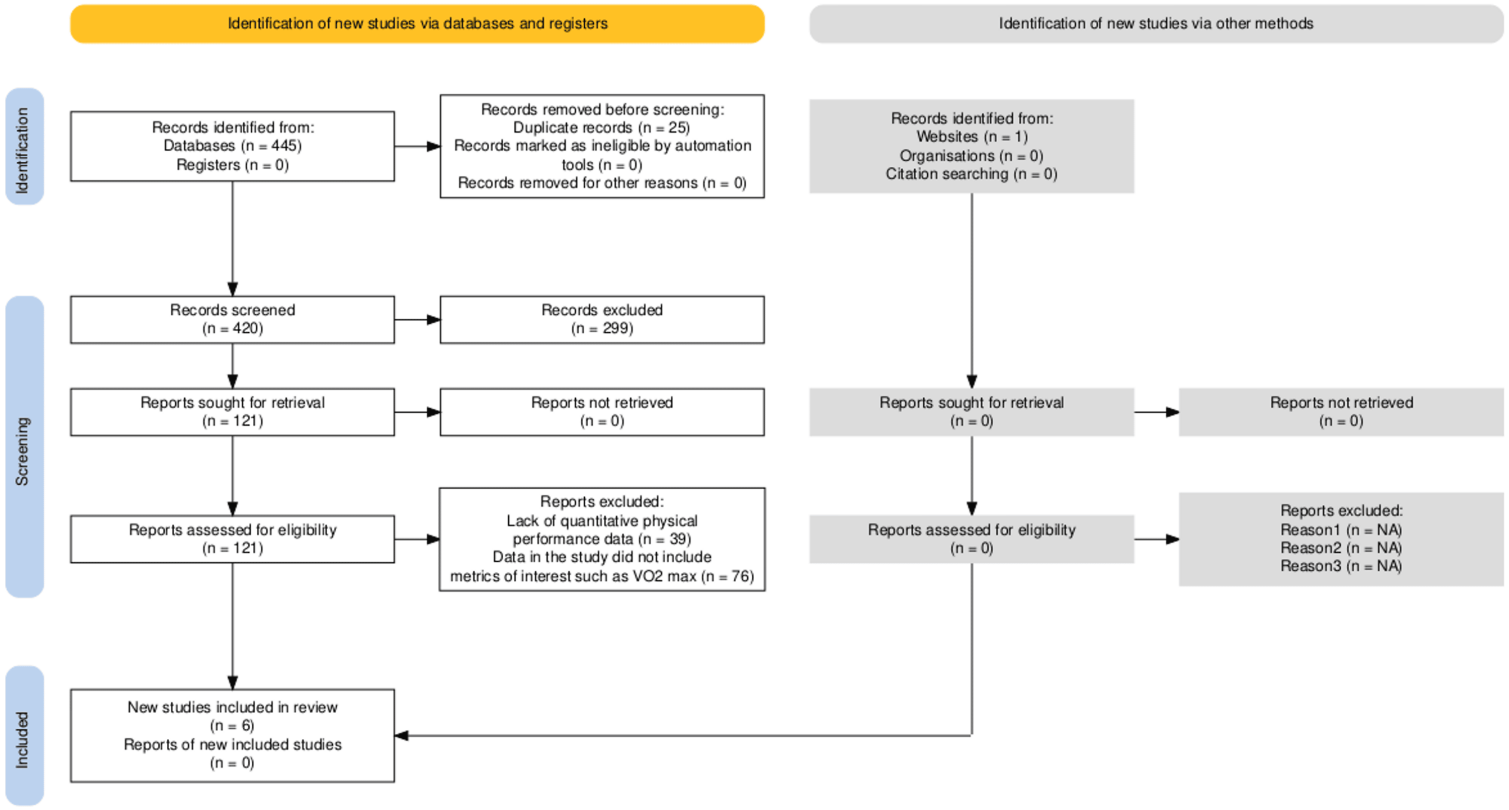
PRISMA flowchart for selection of studies in the systematic review PRISMA: Preferred Reporting Items for Systematic Review and Meta-Analyses Reference [2]

The screening and selection process for the articles included in this review is illustrated in Figure 1. Of the 420 non-duplicate studies, 299 were removed by title and abstract screening. The remaining 121 studies were assessed with full text, and six studies that met the inclusion criteria were selected. All eligible studies were clinical trials with human subjects above the age of 18. They all investigated the use of a low-carbohydrate ketogenic diet in comparison to a high-carbohydrate diet. Finally, they all evaluated and reported a physical performance metric by which the control arm was compared to the intervention arm.

Characteristics of Studies and Subjects

Table 1 below gives a brief overview of the selected studies. The selected studies evaluated a combined total of 98 subjects. Four of the six included studies were geared toward endurance athletes and aerobic exercise, as reflected in the reported data on VO2max and RER. The average duration of the studies was roughly 10 weeks [4-9]. As one study was a crossover clinical trial, the study duration was divided into the intervention and control periods. It is noteworthy that the exercise type varied across the selected articles. One study recruited endurance-trained male cyclists [4]. Another study recruited recreational CrossFit athletes [5]. One study recruited endurance athletes across runners and cyclists [6]. One study recruited Natural bodybuilders [7]. One study recruited military recruits [8], and one study recruited healthy volunteers from the community [9]. All six studies employed a low-carbohydrate ketogenic diet. The investigators either provided the participants with meals or provided guidance on recommended dietary choices. Ketosis was confirmed via serum ketone level measurements, except for one, which monitored participants' adherence via interview [6].

**Table 1:** Characteristics of the studies included in the systematic review HDL: High density lipoprotein, LDL: Low density lipoprotein, CK: Creatinine kinase, LDH: Lactate dehydrogenase, HR: Heart rate, RER: Respiratory exchange ratio, TG: Triglyceride, CKD: Cyclical ketogenic diet, VO2max: Maximum rate of oxygen consumption.

Author, Year, Country	Study type, Sample Population	Selection Pool	Exercise Type	Intervention Period	Data
Zajac et al. (2014), Poland [4]	Randomized Crossover Trial, eight males, Age 28.3 +/- 3.9	Endurance trained male cyclists	Exercise: Cycling, mixed or standard Western diet included 50% carbohydrates, 30% fats and 20% protein, while the ketogenic diet was composed of 70% fat, 15% protein and 15% carbohydrates	Four weeks on Mixed diet, four weeks on ketogenic diet	Triglyceride, HDL, LDL levels, Insulin, Glucose, CK, LDH, Testosterone, and Cortisol level, HR, RER, and VO2max, resting and exercise concentrations of glucose (Glu, mg/dL) (GL 2623), free fatty acids (FFA, mmoL/L) (FA 115) and β-hydroxybutyrate (β-HGB, mmoL/L), with the participation of NAD and β-hydroxybutyrate dehydrogenase, were evaluated
Kephart et al. (2017), USA [5]	Non randomized, Case-Control Study, 12 participants, 31 years old +/- 2 years	Recreational Crossfit Athletes	Exercise: Crossfit training	12 weeks anaerobic perfomance testing	Weekly betahydroxybutarate levels, Body Composition using DXA, Vastus Lateralis Thickness, Fasting glucose, HDL, TG, LDL, VO2 peak
Zinn et al. (2017), New Zealand [6]	Case study design, five participants (4 female, 1 male), age 51 +/- 4	Endurance athletes	Exercise: Endurance exercises; running, cycling	10 weeks, assessment by interview	Interview on experience, sum of skin folds, time to exhaustion, VO2max
Paoli et al. (2021), Italy [7]	Randomized Clinical Trial, 19 volunteers, age 27.4 +/- 10.5	Natural Body Builders	Exercise: Daily workout schedule divided by muscle groups and training sessions aimed at increasing strength and muscle mass	Eight weeks	Body fat, lean mass, triglycerides, glucose, insulin, adherence to the diet and ketonemia were monitored through a portable device
LaFountain et al. (2019) USA [8]	Non-randomized two group prospective study, 29 (26 +/- 8)	Military Subjects	Exercise: Supervised endurance exercise and Calisthenics	12 weeks with concurrent anaerobic performance testing	Resting metabolic rate, RER, Body composition, Blood glucose, Physical Training assessments, Weekly betahydroxybutarate levels
Kysel et al. (2020) Czech Republic [9]	Randomized controlled trial, 25 males, 23 +/- 5	Healthy Volunteers	Exercise: Regular resistance training combined with aerobic training	Eight weeks	Physical training assessments, VO2max, Overall adherence to diet was checked once weekly by a nutritionist. Furthermore, adherence to CKD was evaluated through urinary ketone measurements performed twice daily and by measurement of blood β-hydroxybutyrate at the end of the study.

Effect of Ketogenic Diet on VO2max

Two of the selected studies evaluated VO2max similarly and could be compared via a t-test for statistical analysis [4,9]. Zajac et al. (2014) evaluated performance (including VO2max) in athletes exercising with the Excalibur Sport Cycloergometer. Kysel et al. (2020) utilized spiroergometry. The analysis of VO2max showed that while the ketogenic diet resulted in higher mean values (Keto: 43.0, 59.4; Non-Keto: 38.2, 56.02), the t-test analysis (t(4) = -5.761, tcrit = -12.71, p > 0.05) indicated no significant difference as determined by the acceptance of the null value.

Effect of Ketogenic Diet on Respiratory Exchange Ratio (RER)

Similar to the analysis of VO2max, RER values suggested a slight decrease with the ketogenic diet (Keto: 0.94, 0.79, 0.75; Non-Keto: 0.97, 0.83, 0.83), but the t-test results (t(4) = 3.273, tcrit = 4.303, p > 0.05) were not statistically significant [4,7,8].

Effect of Ketogenic Diet on Bench Press Performance

Bench press performance showed no improvement on the ketogenic diet (Keto: 0; Non-Keto: 6.3, 3.5), supported by t-test results (t(4) = 3.5, tcrit = 12.71, p > 0.05) [8,9].

Effect of Ketogenic Diet on Body Mass

Body mass changes indicated a trend towards weight loss on the ketogenic diet (Keto: -0.88, -7.7, -4.6; Non-Keto: 1.33, 0.1, -4.5), however, the t-test (t(4) = 1.47, tcrit = 4.303, p > 0.05) showed no significant difference [7-9]. Zinn et al. (2017) measured the change in body mass by kg, however, the values from which this data were not published in a way that could be applied to the t-test formula used in this review. The results of our t-test analyses are illustrated in Table 2.

**Table 2:** t-test results VO2max: Maximum rate of oxygen consumption, RER: Respiratory exchange ratio.

Prospective Trials	Secondary outcome	Overall Δ	Tstat at ⍺ 0.05	Tcrit at ⍺ 0.05	Standard Deviation	Null hypothesis
	VO2max					
Zajac et al. (2014)	Non-Ketogenic Diet: 38.2, Ketogenic diet: 43.0	4.8				
Kephart et al. (2017)	Non-Ketogenic Diet: 56.02, Ketogenic diet: 59.4	3.38				
t-test results			-5.761	-12.71	1.0234	accepted
	RER					
Zajac et al. (2014)	Non-Ketogenic Diet: 0.97, Ketogenic diet: 0.94	-0.03				
Paoli et al. (2021)	Non-Ketogenic Diet: 0.83, Ketogenic diet: 0.79	-0.04				
LaFountain et al. (2019)	Non-Ketogenic Diet: 0.83, Ketogenic diet: 0.75	-0.08				
t-test results			3.273	4.303	0.0265	accepted
	Mean Δ in Bench press reps					
LaFountain et al. (2019)	Non-Ketogenic Diet: 6.3, Ketogenic diet: 0	-6.3				
Kysel et al. (2020)	Non-Ketogenic Diet: 3.5, Ketogenic diet: 0	-3.5				
t-test results			3.5	12.71	1.98	accepted
	Δ Body Mass in kg					
Zinn et al. (2017)	data not provided as separate units	-4	omitted	omitted		
Paoli et al. (2021)	Non-Ketogenic Diet: 1.33, Ketogenic diet: -0.88	-2.21				
LaFountain et al. (2019)	Non-Ketogenic Diet: 0.1, Ketogenic diet: -7.7	-7.8				
Kysel et al. (2020)	Non-Ketogenic Diet: -4.5, Ketogenic diet: -4.6	-0.1				
t-test results			1.47	4.303	3.2634	accepted

Discussion

Athletic performance, weight loss, and overall health have been popular topics that have led to the debate over which diets may yield the most benefits to their consumers. The ketogenic diet has risen in popularity and has been speculated to reduce skeletal muscle glycogen levels and stifle muscle anabolism [5]. Some of the more recent studies on nutrition and exercise metabolism have attempted to examine the scientific evidence for the hypothesis that endurance training undertaken with low carbohydrate availability promotes greater adaptive changes compared to high carbohydrate availability. This review investigates the evidence of its effects on performance and body mass as demonstrated by the selected clinical trials. Two of the studies included in this review found a clinically insignificant increase in VO2max post-ketogenic diet consumption [4,5]. Three of the studies demonstrated a slight decrease in RER, which was also statistically insignificant [4,7,8]. Three of the studies demonstrated a statistically insignificant decrease in body mass [7-9]. While there appear to be some trends, this study remains inconclusive as to the extent of the changes. Equally important for those who would seek to use the ketogenic diet as a means to reach personal goals is the tolerability and sustainability of the dietary restrictions. One study of five participants conducted regular interviews with the participants and uncovered experiences such as improved skin health, decreased gas, reduced inflammation, and an improved sense of overall health [6]. The same participants did report a transient feeling of loss of power, and overall, did not enjoy being on the diet long-term. These findings raise the question of whether a ketogenic diet could be employed transiently during specific phases of physical training for optimal effect.

Because the search was conducted in a limited fashion, there are likely more clinical trials with more valuable information on the effects of the ketogenic diet on performance. Also, there is the potential risk of publication bias. It would be challenging to reliably estimate the extent of evidence that may not have been published for various reasons. Overall, we do believe that there is an opportunity to gain a more objective understanding of the physiological effects of the ketogenic diet.

## Conclusions

While multiple articles within the databases suggest clear trends indicating that a ketogenic diet may induce significant physiological and metabolic changes, the precise nature of these changes and their implications remain unclear due to the ongoing scarcity of data in this review. This investigation yields several key learning points. Firstly, conducting a robust systematic review necessitates defining specific parameters for evaluation before commencing the search. Unfortunately, our study initially searched and then selected parameters based on the obtained results. Had we predetermined each measure individually, such as correlating the ketogenic diet with VO2max, our data might have demonstrated greater strength. Secondly, relying on only one to two data samples severely restricts the degrees of freedom in a t-test, potentially compromising the significance of the results.

Despite these limitations, notable trends within the studies include weight loss, reduction in fat percentage, increased VO2max, and decreased RER. The implications of these findings for athletic performance remain uncertain, necessitating further comprehensive investigation to obtain additional data. This review ultimately highlights the limitations of the current body of research, including small sample sizes and heterogeneity in study methodologies, which constrain the ability to draw definitive conclusions. The findings suggest potential physiological and metabolic benefits of a ketogenic diet, such as weight loss and increased VO2max, but emphasize the need for further research with larger, more homogeneous samples and more targeted investigations to confirm these potential advantages.
